# The airway mycobiome and interactions with immunity in health and chronic lung disease

**DOI:** 10.1093/oxfimm/iqae009

**Published:** 2024-08-22

**Authors:** Orestis Katsoulis, Oliver R Pitts, Aran Singanayagam

**Affiliations:** Centre for Bacterial Resistance Biology, Department of Infectious Disease, Imperial College London, London SW7 2DD, UK; Centre for Bacterial Resistance Biology, Department of Infectious Disease, Imperial College London, London SW7 2DD, UK; Centre for Bacterial Resistance Biology, Department of Infectious Disease, Imperial College London, London SW7 2DD, UK; National Heart and Lung Institute, Imperial College London, London SW7 2DD, UK

**Keywords:** chronic lung disease, fungi, mycobiome, immunity

## Abstract

The existence of commensal fungi that reside within the respiratory tract, termed the airway mycobiome, has only recently been discovered. Studies are beginning to characterize the spectrum of fungi that inhabit the human upper and lower respiratory tract but heterogeneous sampling and analysis techniques have limited the generalizability of findings to date. In this review, we discuss existing studies that have examined the respiratory mycobiota in healthy individuals and in those with inflammatory lung conditions such as asthma, chronic obstructive pulmonary disease and cystic fibrosis. Associations between specific fungi and features of disease pathogenesis are emerging but the precise functional consequences imparted by mycobiota upon the immune system remain poorly understood. It is imperative that further research is conducted in this important area as a more detailed understanding could facilitate the development of novel approaches to manipulating the mycobiome for therapeutic benefit.

## Introduction to the mycobiome

The human microbiota comprises an extensive ecosystem of trillions of microorganisms that are known to colonize mucosal sites within the body including the skin, gut, and urogenital tract. Although the lower airways were traditionally believed to be a sterile environment, advances in molecular microbiological techniques and sequencing technologies have facilitated the characterization of the presence of microorganisms, which are now understood to be commensal inhabitants of respiratory mucosal surfaces [1]. Termed airway microbiota, this community of commensal microorganisms is populated by complex networks of transient and colonizing bacteria, fungi, and viruses, which is unsurprising when one considers that the human respiratory tract represents the primary point of entry for countless microorganisms, especially through airborne particles.

The fungal components of this microbial ecosystem are termed mycobiota, or the mycobiome, where the former refers to the microorganisms themselves and the latter to their corresponding genomes [2]. Body sites that are recognized to harbour a mycobiome include the oral cavity, gastrointestinal (GI) tract, respiratory tract, urogenital tract, and the skin [3]. The composition of human mycobiota displays significant interindividual and intraindividual variability [4]. The shaping of the human mycobiome begins immediately after birth and may be affected by a range of factors such as birth weight, gestational age/delivery and early feeding methods [5, 6]. In adulthood, the diversity of the mycobiota is further influenced by age, sex, diet, use of antibiotics or antifungals, and environmental factors [3, 7].

To date, most studies of the pulmonary microbiome have focused on the presence and functional roles of bacterial communities. Commensal bacteria have been shown to actively contribute to airway homeostasis and determine the development and function of the local immune system through their capacity to influence immune cell maturation, produce antimicrobial molecules and balance pro- and anti-inflammatory responses [8]. The bacteriome can be influenced by several factors, such as diet, age, environment, genetics, and antibiotic use. These external influences can induce perturbations leading to dysbiosis, an imbalance in the composition and function of the microbiome. Various chronic diseases have been linked to microbial dysbiosis with implications for the pathogenic progression of these illnesses [9, 10].

More recently, it is becoming apparent that, similarly to bacterial communities, mycobiota play key roles in maintaining host homeostasis. However, our understanding of the key commensals and associated mechanisms remains limited. Although modest in number compared to other classes of resident commensals (representing approximately 0.1% of microorganisms in the body), the massive genomes of fungi could convey significant implications for the extent of their influence and role as keystone species in the airways [11]. Indeed, certain fungal species are being implicated in chronic respiratory diseases (CRDs) where dysbiosis is likely to have an impact upon cardinal disease features such as chronic inflammation, defective mucociliary clearance and immunosuppression [12, 13].

In addition to the apparent importance of the mycobiome in healthy homeostasis, opportunistic members of the mycobiome also have the potential to transcend from commensalism to pathogenicity, a phenomenon which is highly dependent upon altered host immunity, microbial dysbiosis and environmental factors [14, 15]. Importantly, fungal pathogens exhibit remarkable adaptability to the human lung, partly enabled by the abundance of biosynthetic gene clusters in these organisms, which produce bioactive secondary metabolites that include human toxins (e.g. aflatoxin) [16]. *Aspergillus fumigatus*, the most frequently isolated colonizer in humans, represents a prime example with over 30 biosynthetic gene clusters [17, 18]. Studies are also beginning to highlight symbiotic and antagonistic trans-kingdom relationships between bacterial and fungal commensals. The presence of certain microbial taxa has implications for this switch to pathogenicity. It is therefore crucial to understand the complex biological synergy between fungi, bacteria and also viruses with the host immune response, to fully decipher the complex multifaceted interactions that seemingly govern airway homeostasis.

In healthy people, inhaled spores are expelled from the airways through the process of mucociliary clearance [19]. However, a variety of factors including, but not limited to, a deficient immune response, presence or absence of CRD, and external factors such as smoking or pollution, can lead to the perturbation of mycobiota in the airways of patients, thus increasing the likelihood of pathogenic fungal colonization and infection.

## Challenges in studying the mycobiome

Research around the respiratory mycobiome faces several challenges, both inherent to the microorganisms being interrogated and teething problems from the burgeoning nature of the field. Firstly, there are approximately three million species of fungi and fungus-like organisms, making them the second biggest group of eukaryotes based on global diversity [20]. Furthermore, compared to other eukaryotes, fungi have simple cellular structures, often encompassing morphologically ambiguous structures, which poses challenges in accurate identification [20]. Moreover, traditional culture-based classification techniques do not provide an authentic representation of respiratory mycobiota due to the large proportion of non-culturable species, biases towards faster growing species or even masking of rarer, morphologically similar species [21, 22]. With the advent of high throughput amplicon sequencing and shotgun metagenomics came greater power to more accurately discern the spectrum commensals that reside in the airways. However, technical obstacles pertaining to these techniques remain. For example, different DNA extraction methods have been suggested to influence bacterial and fungal community composition and contamination with environmental microorganisms in low biomass airway samples remains a major concern [22, 23]. The lack of suitable marker genes limits operational taxonomic unit (OTU) classification and accurate determination of relative abundance. Typically, methods target specific genes encoding internal transcribed spacer (ITS)1, ITS2 and 18S rRNA, however the complimentary primers for these sequences produce variable results and can amplify the genes of other eukaryotes [24]. Moreover, intragenomic variation in the ITS sequences in DNA barcoding methodologies and a lack of sufficient and suitable reference databases for fungal identification also hinders comprehensive categorization of respiratory mycobiomes [25, 26] with the existing databases (e.g. FungiDB, Mycobank, Ensembl Fungi) having limitations in terms of coverage of the full spectrum of fungal taxa. Finally, there is a lack of standardization between studies owing to heterogeneity in design, sampling, sequencing processes and the bioinformatic pipelines and analyses used.

Holistically, these limitations have led to inconsistencies between studies carried out by different groups. Most tend to agree at the phylum level; however, differences arise at the genus level, which along with the large interindividual mycobiome variation, results in a lack of uniform consensus regarding typical composition. Delving into the challenges of fungal species identification in great depth is beyond the scope of this review and we would point the reader towards recent publications by Tiew *et al.* [27] and Bharti *et al.* [28] for more extensive elaboration. Nevertheless, it is evident that a consensus on how to design and conduct studies for the characterization of the human airway mycobiome needs to be reached, to improve reproducibility and standardized comparison of results.

## Airway mycobiome in health

Mycobiome composition in the respiratory tract is under constant flux with evolution from initial colonization at birth through childhood and adulthood. Several factors within the categories of vertical (from the mother) and horizontal (environmental) transmission modulate the spectrum of fungal microorganisms that inhabit the airways [29, 30]. These include: the delivery method, gestational age and later the feeding method, while diet continues to be a predominant factor throughout adulthood, along with weight and geographical location [30]. The potential of transient mycobiota to establish long-term colonization in the airways can vary by species, microbial burden, and the pulmonary microenvironment of the host [31, 32]. Further variation between individual mycobiome profiles derives from the environmental niches found in the pulmonary ecosystem, arising from differences in mucus or surfactant secretion, gene expression, pH, and nutrient or oxygen availability [33].

Although ecosystem composition tends to overlap, the respiratory mycobiome can be split into two interconnected regions—the upper and lower respiratory tract (URT and LRT). Previous studies on the respiratory mycobiome have been limited to focussing on potentially pathogenic fungi, which accounts for the fact that, until recently, fungal colonization of the respiratory tract was believed to be transient [3]. More recent investigations have provided evidence that the respiratory tract is permanently populated by fungi, with specific genera and species preferentially colonizing the airways over the oral cavity [3, 34]. The majority of fungi identified in the human respiratory tract reside in the *Basidiomycota* and *Ascomycota* phyla, with the most commonly identified genera in lung tissue being *Cladosporium*, *Eurotium*, and *Aspergillus* [34, 35]. Importantly, the gut mycobiome has been suggested to influence the oropharyngeal and respiratory mycobiome via micro-aspiration [35], while other determinants such as geographic and climate variability, genetics, and environmental factors, have also been hypothesized to play a role in composition variability [11]. The gut-lung axis is well established in the context of the bacteriome and roles for interactions between gut and lung mycobiota are also emerging [36].

### Upper respiratory tract

The oropharyngeal mycobiome is important to consider as it represents a major pathway for transmission to the respiratory tract through micro-aspiration. The microbial communities of the oral cavity are amongst the most diverse in the human body and they exhibit huge interindividual variation [37]. Composition constantly develops and evolves over the course of a lifetime, with colonization starting at birth and progressing with age [38]. The prevailing consensus on the oral mycobiome is an ecosystem dominated by *Candida*, *Cladosporium*, *Aureobasidium* and *Aspergillus*, with a notable presence of *Fusarium*, *Penicillium* and *Cryptococcus* [39–41]. In the adult oral mycobiome, most colonizers belong to the phyla *Ascomycota*, *Basidiomycota*, *Glomeromycota* and *Mucoromycota* [42, 43].

One of the first studies to investigate the oral mycobiome was by Ghannoum *et al.* [40]. This pioneering work examined 20 individuals and identified 85 species of fungi (74 culturable, 11 non-culturable) by multi-tag pyrosequencing, a predecessor to NGS, making it the first to use such technology to this end. Subjects were found to have a range of 5 to 39 fungal genera, and a core set comprising of *Candida* (75%); *Cladosporium* (60%); *Aureobasidium* (50%); *Aspergillus* (35%); *Fusarium* (30%), and *Cryptococcus* (20%) was identified. Further studies refined this ‘normal’ composition, notably Dupuy *et al.* who used an improved pyrosequencing approach to overcome process-induced sequencing errors and accurately assign fungal taxonomy, reporting the significant presence of *Malassezia* in saliva samples from all six participants [43]. It is now recognized that two distinct oropharyngeal mycobiota profiles exist—one dominated by *Candida* and the other by *Malassezia*. *Candida* sp. thrive in the low pH environment of the oral cavity and is the only genus to have been shown to reach a significant biomass, being associated with a distinct oral ecology [44]. Indeed, mechanistic animal studies have highlighted synergistic interactions with acidogenic bacteria, and the presence of *Candida* has been significantly correlated with caries including tooth loss, periodontitis and expansion of *C. albicans* in oral candidiasis [44–46].

Numerous ITS sequencing studies have suggested that *Candida*, *Malassezia*, *Penicillium*, *Cladosporium*, *Pichia*, *Alternaria*, *Aspergillus*, *Cryptococcus*, *Trichosporon* and *Rhodotorula* are high abundance genera which can be more easily isolated from oral samples, implying they are likely the main fungal colonizers [40]. Furthermore, *Pichia* have been shown *in vitro* to inhibit the growth of potentially pathogenic *Candida*, *Aspergillus* and *Fusarium* [47]. It is important to recognize that there are additional challenges associated with identification of fungal taxa in the oral mycobiome, as it represents the immediate region post ingestion of food or fungal spores from the environment. Thus, careful assessment of which species are colonizers and which are transient is required. Despite these limitations, ITS sequencing suggests that *Candida*, *Pichia*, and *Fusarium* species are common oral colonizers [33, 40].

### Lower respiratory tract

**Figure 1.**
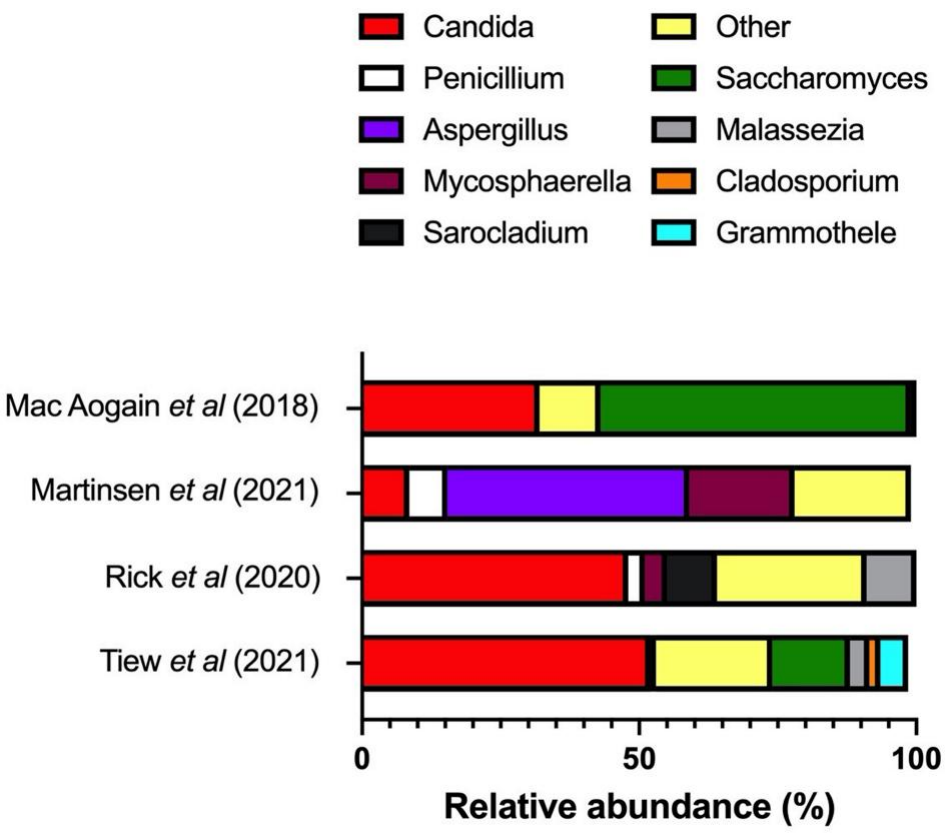
A plethora of fungal genera inhabit the lungs but there is little in the way of composition consensus between recent studies. The different genera and their relative abundances identified from recent sequencing studies. These are mainly from analysis of airway samples from small groups of subjects with CRDs. Research articles referenced: Mac Aogain *et al*. [48], Martinsen *et al*. [49], Rick *et al*. [50], Tiew *et al*. [51]

Compared to the oral mycobiome, the lower respiratory tract is even more poorly characterized with a very small number of studies to date focussing on pulmonary mycobiomes in subjects with chronic respiratory diseases. Figure 1 summarizes the different genera and their relative abundances identified from recent sequencing studies.

Up until 2015, the existence of a distinct, resident mycobiome in the lungs of immunocompetent individuals was not recognized [34]. Indeed, fungal colonization of the lungs can be difficult to approach due to its potentially stochastic nature, giving the appearance of transience when colonization has in fact occurred [2, 34]. The precise diversity of fungal species that inhabit the lungs is likely governed by environmental exposure to filamentous fungi and yeasts, micro-aspiration from the oropharynx or by direct inhalation of spores [3].

One of the initial studies assessing the lower respiratory mycobiome was conducted by Van Woerden *et al.*, who carried out 18s pyrosequencing of DNA extracted from sputum samples in 13 non-atopic healthy subjects [52]. In total they identified 136 fungal taxa in the healthy lungs, with the species *Eremothecium sinecaudum*, *Systenostrema alba*, *Cladosporium cladosporioides*, *Vanderwaltozyma polyspora* and *Entophlyctis helioformis* being most prominent. These findings were further developed in a study by Cui *et al.* where 18s and ITS sequencing were performed on oral wash, induced sputum and bronchoalveolar lavage (BAL) collected from 56 individuals [34]. The authors demonstrated clear patterns of shared and unique taxa between oral wash and BAL communities. Several fungal species were identified as predominant in the lower respiratory tract including *Ceriporia lacerata*, *Saccharomyces cerevisiae*, and *Penicillium brevicompactum*.

One of the major limitations with the current pool of literature on mycobiome composition in the lower respiratory tract is that our understanding is largely based on analysis of sputum samples. Prior studies examining the bacteriome indicate overlap between oropharyngeal and lower respiratory microbiome profiles in sputa [53]. Sputum is thus less adequate for assessing fungi that may be present in more distal lung tissue. Therefore, a major future priority for the field is to build an anatomic catalogue or atlas of respiratory tract mycobiota through lower airways (e.g. BAL) sampling. Notably, studies that have employed lower airways sampling have shown greater prominence of *Candida* and *Aspergillus* [49, 50].

## Interactions of the mycobiota with the host immune response

We remain within the infancy of our understanding of how respiratory mycobiota may influence pulmonary host immunity, although inferred evidence of these potential interactions can be gained from studies of other mucosal sites such as the gastrointestinal tract. Structural ligands and metabolites from bacteria, viruses, and fungi of the lung microbiota have a range of functional effects upon innate and adaptive immunity, highlighting how these components can mechanistically influence the development and function of the immune system. While the interaction between bacterial pathogen-associated molecular patterns (PAMPs) and host pattern recognition receptors (PRRs) such as Toll-like receptors (TLRs) is well-established [54, 55], our understanding of fungal PAMPs and their interactions and downstream effects upon host immunity remains more limited. Nevertheless, studies are beginning to indicate that commensal fungi, similar to bacteria, provide basal stimulation to the immune system. This may contribute to protection against pathogens, as well as maintaining tolerance towards ‘helpful’ members of the microbiota [15]. Jiang *et al.* provided evidence that susceptibility to colitis and influenza A infection in gut microbiota-depleted mice, could be reversed by mono-colonization of the gut with *C. albicans* and *S. cerevisiae* with protective effects induced by stimulation by the fungal cell wall component mannans [56].

Moreover, as with commensal bacteria, metabolite synthesis by mycobiota may also be a major mechanism of immune modulation. Differential host sensing of fungal determinants from various species can significantly impact homeostatic immune modulation [56, 57]. Metabolites produced by *Malassezia,* including malassezin and indolo [3,2‐*b*] carbazole, are known to act as potent ligands for the aryl hydrocarbon receptor (Ahr), leading to downstream signalling that is vital for epithelial tissue repair, barrier homeostasis and immune cell development [57, 58]. Furthermore, *Malassezia* produce lipases that catalyse the conversion of host triglycerides in the skin into short-chain fatty acids (SCFAs), metabolites with pleiotropic immunomodulatory effects that have been well-documented in numerous studies [59–61]. *Aspergillus fumigatus* can also induce production of short-chain fatty acids through biodegradation of wheat straw lignin [61] but whether these processes occur in the human gut or lungs is unknown. Importantly, a recent study illustrated that the metabolites produced by microorganisms need not necessarily exert their effects exclusively on their local milieu. Specifically, the researchers showed that mice suffering from gut dysbiosis with an overgrowth of *C. albicans* were more likely to suffer from allergic airway inflammation [60]. The authors determined that this was mediated by an increase in the secretion of prostaglandin E_2_ (PGE_2_) by *C. albicans*, which resulted in the polarization of alveolar macrophages towards the alternatively activated M2 inflammatory phenotype. This is a prime example of fungal metabolite translocation to the lungs being a key component of airway disease pathogenesis. The diversity of other similar metabolites produced by the mycobiota and the extent to which they influence the host immune response, both within and beyond the airways, remains to be investigated.

Factors such as disruption of the healthy microbiota, defects in host barrier functions and immunodeficiency allow fungi such as *C. albicans* and *A. fumigatus* to transition from commensalism to pathogenicity. These opportunistic fungal pathogens have been shown to modulate the human immune system, both to the benefit and detriment of the host. Pre-exposure of monocytes to *C. albicans* primed these cells towards a pro-inflammatory phenotype, leading to increased secretion of IL-6 and TNF-α upon stimulation with TLR ligands, an effect that could be recapitulated by inoculation with β‐1,3‐glucan alone, which is detected by the receptor dectin-1 [62]. Whether or not this immune ‘training’ effect is replicated *in vivo* by resident *C. albicans* of the mycobiota, and how it is balanced with the requirement to dampen the immune response and remain tolerant against commensal members of the microbiota, is yet to be determined.

Control of the host immune system is where fungi seek to attenuate immunological responses. For example, *C. albicans* has been found to preferentially stimulate the TLR2 receptor, inducing a Th2 immune response, allowing it to persist in the absence of a potent pro-inflammatory Th1 response [63]. It also synthesizes farnesol, which significantly reduces cytokine production by macrophages [64]. Meanwhile conversion of tryptophan to kynurenine by *A. fumigatus* through host enzyme indolamine 2,3-dioxygenase results in immunosuppression [65, 66].

As discussed, it is important to note that many of the aforementioned findings derive from studies investigating the gut mycobiome. Therefore, future studies should be directed at examining whether these findings would be more specifically applicable to respiratory fungal commensals specifically residing within the pulmonary microenvironment.

## Interactions between fungal and bacterial commensals

In addition to directly influencing the host immune response, members of the microbiota can also indirectly impact host immunity by interacting with each other [67–69]. This is especially prominent in the gut and the lower airways, where fungi and bacteria of the microbiota coinhabit the same environmental niches in polymicrobial biofilms attached to mucosal surfaces [70, 71]. These mixed biofilm environments can convey several benefits to the cohabitants, including metabolic cooperation and evasion of immune and anti-microbial agents [71]. Indeed, the interplay between fungi and bacteria can be bidirectional, with mechanical and chemical interactions shown to selectively affect the growth and survival of different species, depending on the specific environmental conditions [72]. For instance, several mutualistic and non-mutualistic competitive interactions between *P. aeruginosa* and *A. fumigatus* have been recorded in the context of co-infection in patients with cystic fibrosis. Mechanisms include inhibition of biofilm formation and growth suppression via nutrient sequestration or reactive oxygen species (ROS) production [72–74]. It is also possible for fungal-bacterial interactions to enhance the virulence of pathogens, either directly, through the activity of metabolic products [75], or indirectly, through the incorporation of pathogens into biofilms, hampering the activity of the immune system and antimicrobials [76].

Another form of communication between microorganisms that has garnered attention over the past few years is quorum sensing (QS). This phenomenon describes commensal fungi dictating gene expression within bacteria and vice versa [77]. This dynamic form of communication relies on the accumulation of density-dependent small diffusible molecules that transmit a signal upon reaching a specific threshold, highlighting the significance of diversity and population numbers in the composition of the healthy microbiota [78, 79]. Although initially believed to be exclusive to bacteria, QS has been shown to be especially important in fungi as well, where it can regulate the transition from spherical to hyphae form, apoptosis and pathogenicity [78]. Interestingly, the LasIR quorum-sensing system has been implicated in inhibiting *A. fumigatus* biofilms formation, while the QR-regulated toxins pyrrolnitrin and pyocyanin have been shown to inhibit the growth of certain fungi.

## Role of respiratory mycobiota in chronic respiratory diseases

Chronic respiratory diseases (CRDs) such as chronic obstructive pulmonary disease (COPD), cystic fibrosis (CF) and asthma affect approximately 1 billion people worldwide, creating an immense financial burden for healthcare systems globally [78]. These diseases are broadly characterized by chronic airway inflammation and defective mucociliary clearance which may predispose to fungal colonization and infection. This may be worsened by the frequent use of immunosuppressive (e.g. inhaled corticosteroid) and antimicrobial therapies. There is a growing body of evidence indicating that the respiratory mycobiome may play a key role in the etiopathogenesis of CRDs, mainly through inter-microbial and/or host interactions [27]. Several studies have shown that differences in composition and diversity of lung mycobiota between healthy and diseased populations can influence CRD progression, morbidity and clinical outcomes [36]. *A. fumigatus* represents a prime example of this, since its identification by culture in airway samples from subjects with CRDs has been associated with increased clinical severity and higher risk of exacerbation [80, 81]. The following sections will highlight disease-specific alterations in respiratory mycobiota composition within a selection of the common lung diseases COPD, asthma, and CF, and discuss how these perturbations may impact upon progression, severity and management of each disease.

### COPD

Chronic obstructive pulmonary disease (COPD) is a smoking-related progressive respiratory condition marked by a persistent inflammatory response in the lower airways, resulting in irreversible airflow obstruction. Several mechanisms contribute to the development of COPD, encompassing an imbalance between proteolytic and anti-proteolytic activities, mitochondrial dysfunction, infiltration of inflammatory cells into the lungs, and oxidative stress [82]. Key features of COPD include impaired mucociliary clearance, dysregulation of innate immune responses (e.g. type-I interferon) and chronic inhaled corticosteroid use, all of which could theoretically increase the likelihood of fungal dysbiosis [83–85].

To date, a few studies have attempted to characterize the differences between the lung mycobiome profiles of COPD patients and healthy individuals. Martinsen *et al.* profiled the oral and lung mycobiome in patients with COPD compared to healthy individuals but surprisingly found no significant differences between these groups with the most abundant genera being *Candida*, followed by *Malassezia*, *Penicillium* and *Aspergillus* [49]. Conversely Tiew *et al.* reported increased fungal α-diversity in COPD compared to health [51]. Specifically, COPD patients with expansion of *Saccharomyces* within the respiratory mycobiome exhibited increased respiratory symptoms. Subjects with a ‘high-risk’ mycobiome profile dominated by *Aspergillus, Penicillium*, and *Curvularia* dominance had more frequent exacerbations and greater mortality. A subset of these ‘high-risk’ patients also correlated with a sensitization response to these fungi, suggesting that mycobiota can be linked to a measurable host immune response. Importantly, as this study took participants from Singapore, Malaysia and Scotland, the authors were also able to determine, via linear discriminant analysis, that the airway mycobiome in COPD illustrates geographic variation. The differences between this study and the study by Martinsen *et al*. [49], conducted in Norway could thus partially be explained by differences in geographic location. This should be carefully considered when drawing conclusions from studies of this nature. Su *et al.* conducted a longitudinal study of six patients with acute exacerbations, showing that alterations in mycobiome composition occur during the episodes but consistent patterns could not be elucidated from this small study [86]. Similarly, Enaud *et al.* reported that individuals with acute exacerbations exhibited lower α-diversity than stable-state, suggesting expansion of certain fungi towards a pulmonary mycobiome dominated by fewer taxa [87].

Several studies support that *Aspergillus* spp. are detectable and potentially important during exacerbation. Bafadhel *et al.*, observed that approximately 50% of stable patients with COPD at baseline had culturable filamentous fungi, 75% of which were *A. fumigatus* [88] with positive culture for *A. fumigatus* detected in 28% of exacerbations. In another study, 1.3–39% of hospitalized COPD patients developed invasive aspergillosis [89]. Aspergillus colonization is strongly associated with corticosteroid use [90] and may promote airway inflammation, airways hyper-responsiveness and relapse of acute exacerbation [91–93]. Moreover, *Candida* spp. isolation in the lower respiratory tract is associated with increased risk of recurrent exacerbations in COPD [94].

Concomitant infections with *A. fumigatus* and *P. aeruginosa* have also been shown to be a key risk factor for exacerbation, again highlighting the importance of inter-microbial relations [95]. Liu *et al.* reported simultaneous characterization of sputum bacterial and fungal microbiome in 84 stable COPD and 29 healthy subjects, identifying an inverse correlation between bacterial and fungal diversity [96]. Perturbed bacterial-fungal interactions were associated with enhanced pro-inflammatory cytokine (IL-6, IL-8) expression and enrichment of fungal taxa with loss of bacterial commensals identified as a driver of exacerbation susceptibility.

Increased airway eosinophilic/type 2 inflammation is recognized to occur in around 10–30% of patients with COPD [97]. Eosinophilic COPD is associated with an altered mycobiome composition compared to non-eosinophilic profiles with lower α-diversity and higher relative abundances of *Aspergillus*, *Bjerkandera* and *Cladosporium* [98]. These taxa may play key roles in the pathogenesis and progression of COPD with *Bjerkandera* for example being shown to induce eosinophilic infiltration and type-2 inflammatory cytokine expression [99, 100]. *Cladosporium* spp. are known to cause allergic inflammation, airway hyperreactivity and remodelling in mice [101]. Allergic sensitization to *Aspergillus fumigatus* is well recognized in a subset of patients with COPD and associated with a more severe clinical phenotype [102].

Such sensitization is not unique to *Aspergillus fumigatus* however as other fungi including *Alternaria alternata*, *Schizophyllum commune*, *Aspergillus tamarii* and *Rhizopus* spp. have been shown to be sensitizing agents in COPD [102]. It is imperative therefore to consider that clinical symptoms could be attributable to fungi other than *Aspergillus*.

*Pneumocystis* is another key genus that has been identified as potentially important in COPD at stable-state and during exacerbation. In a small Colombian study, a colonization frequency of 32.3% was observed for *P. jirovecii*, with its presence significantly correlating with more severe disease (GOLD stage IV) [103]. Morris *et al*. reported *Pneumocystis* colonization rates of 37% in severe COPD [104], which has also been linked to augmented Th1 inflammatory gene expression [105]. At exacerbation, *Pneumocystis* spp, has also been identified in 11/58 patients (19%) with colonization being shown to be associated with higher serum IL-17 and CD26P levels [106]. Using a sensitive LAMP assay, Xue *et al.* reported increased *P. jirovecii* colonization at exacerbation (67%) versus stable state (43%) [107].

In summary, perturbation of the mycobiome is a recognised feature of COPD with correlative evidence to indicate that these commensals may play key roles in pathogenesis, exacerbation susceptibility and disease progression. Although a wealth of descriptive data exists, the next step will be to utilize reductionist cellular and animal models to decipher functional roles played by fungal commensals. These models are currently lacking and will require future development and refinement.

### Asthma

Asthma is a CRD characterized by airway inflammation, remodelling and airway hyperresponsiveness upon exposure to allergens that results in repeated episodes of heightened symptoms [108]. Disease progression displays great heterogeneity by virtue of the complex interplay between genetic and environmental factors that define susceptibility to clinicopathological features [109]. The role of fungi as a complicating factor in asthma has long been recognized with allergic bronchopulmonary aspergillosis (ABPA), where an allergic airway response develops to inhaled *Aspergillus*, estimated to affect around 11% of severe asthmatics [110]. Detailed discussion about ABPA in the context of asthma is beyond the scope of this review and we point the reader to dedicated reviews on this topic [111].

The relatively small number of studies comparing the mycobiomes of healthy and asthmatic subjects have reached variable conclusions regarding the abundances of different fungi at the genus and/or species level, which could be attributed to differences in sample type, sampling method or sequencing techniques. Findings regarding the α-diversity (measurement of microbiome diversity applicable to a single sample) of the mycobiome in sputum samples from asthma patients have also been conflicting, where some studies suggest an increase and others a decrease of α-diversity in asthmatics compared to healthy subjects [50, 112].

In terms of associations of specific fungi with disease severity and progression, sensitization with *Aspergillus sp.* has been linked, on multivariate analysis, to exacerbation frequency and greater corticosteroid requirement in severe asthma [113]. These observations have been corroborated by mycobiome analysis studies, which found a higher fungal load among patients with asthma compared to healthy controls, with *A. fumigatus* complex accounting for the biggest part of this increase [114]. Moreover, in a study using bronchoalveolar lavage fluid (BALF) samples, an increased abundance of *Aspergillus* along with *Fusarium* and *Cladosporium* was noted in patients with heightened type 2 immune responses [115]. Conversely, in a study utilizing ITS2 sequencing in oropharyngeal swab samples, *Aspergillus* and *Candida* were reported to be more abundant in healthy subjects compared to asthma patients; the opposite was observed for *Malassezia* [116]. Upper airway colonization with *Malassezia* has also been shown to be associated with lower risk of progression to severe exacerbation in asthma [117]. Finally, Sharpe *et al.* identified an important association between increased asthma exacerbations and the presence of *Cladosporium*, *Alternaria*, *Aspergillus*, and *Penicillium* in samples collected at home from patient asthma, which hints at a link between fungal exposure and exacerbation susceptibility [118].

Fungal and bacterial dysbiosis in the human gut microbiome has also been implicated in the immunopathology of asthma. Early studies utilizing a mouse model of gut microbiota dysbiosis and intestinal overgrowth of *C. albicans*, reported the induction of a type 2-driven allergic airway response upon exposure to *A. fumigatus*, compared to control mice with an intact microbiota [119]. A similar mouse model was used by Kim *et al.* to elucidate a mechanism of promotion of type 2 immunity through M2 polarization of alveolar macrophages via fungi-produced PGE_2_ [60]. Although detailed discussion about the gut mycobiome is outside the scope of this review, the importance of these studies lies in highlighting the principle of fungal commensals to modulate the host immune system. Similar mechanisms are likely to occur in the pulmonary environment, thus emphasizing the need for further research.

In summary, the mycobiome is likely to play an important role in asthma, particularly in relation to type 2 inflammatory pathways that characterize this disorder. More detailed functional insight will be required to determine the key fungi and mechanisms involved.

### Cystic fibrosis

Cystic fibrosis (CF) is a monogenetic disorder affecting approximately 100 000 people worldwide, associated with decreased life expectancy and a huge treatment burden for patients [120]. It is caused by autosomal recessive mutations in the CF transmembrane conductance regulator (CFTR) gene, which encodes an epithelial transmembrane protein that primarily functions as a chloride channel [120]. In the pulmonary microenvironment, loss of CFTR function results in an electrolyte imbalance, leading to the secretion and accumulation of abnormally thickened mucus that compromises the airway lumen and impairs mucociliary clearance. This culminates in chronic microbial colonization of the lower airways [121]. Recurrent and chronic respiratory infections trigger vigorous inflammatory responses that lead to pulmonary tissue destruction and progressive loss of function, representing the principal cause of loss of quality of life and decreased life expectancy [60].

Although colonizing bacterial pathogens such as *P. aeruginosa*, *S. aureus* and *H. influenzae* are well-recognized to be associated with pulmonary exacerbations, heightened inflammation and mortality in CF patients, far less is known about fungi. Delhaes *et al.* performed the first characterization of the respiratory mycobiome in CF patients, reporting lower α-diversity of both bacteria and fungi in patients with decreased lung function and poor clinical status, and highlighting *C. albicans* and *A. fumigatus* as particularly abundant colonizing species [122]. Subsequent studies have shown a variety of results in terms of defining the prevalence of fungi in the respiratory environment of CF patients, with this variation being mainly attributed to geographical and environmental factors or differences in study design [123]. Nevertheless, the majority of studies agree that *Candida* frequently dominates respiratory fungal communities within CF, with *C. albicans*, *C. parapsilosis*, and *C. dubliniensis* representing the most commonly encountered species. *Malassezia* and *Aspergillus* species have also been frequently identified as colonizers of the lower respiratory tract (LRT), likely due to micro-aspiration events [124–127].

*Aspergillus* has been the subject of much focus in CF and is detectable in around 10–50% subjects [128]. *A. fumigatus,* has been reported to increase with patient age and to be associated with disease severity, as well as corticosteroid and chronic antibiotic use [128–130]. As in asthma and other respiratory conditions, some of the major problems associated with chronic *Aspergillu*s colonization include *Aspergillus* bronchitis, *Aspergillus* sensitization and ABPA. The latter in particular is an allergic inflammatory response process to fungal elements that results in airway remodelling and obstruction and is a frequent cause of pulmonary morbidity in CF [130]. The prevalence of *A. fumigatus* has been reported to increase with patient age, and to be associated with disease severity and chronic antibiotic use [126]. Accordingly, it has been speculated that the composition of the CF respiratory microbiota is subject to an array of dynamic selective pressures dependent on the disease stage, ranging from nutrient availability, pH, oxygen pressure, use of antibiotics, host immunity, and inter-microbial influences. For further information on Aspergillus in CF, we point the reader towards recent review articles dedicated to this topic [128].

Major fungal commensals that are expanded within the airway mycobiome of CRDs are summarized in Table 1.

**Table 1.** Summary of fungal commensal taxa that have been shown to be increased within the airway mycobiome in COPD, asthma and cystic fibrosis

Disease	Increased within airway mycobiome
COPD	Aspergillus Candida Penicillium Cladosporium Bjekandera Pneumocystis
Asthma	Aspergillus Fusarium Cladosporium Malassezia
Cystic Fibrosis	Aspergillus Candida Penicillium Scedosporium

**Figure 2.**
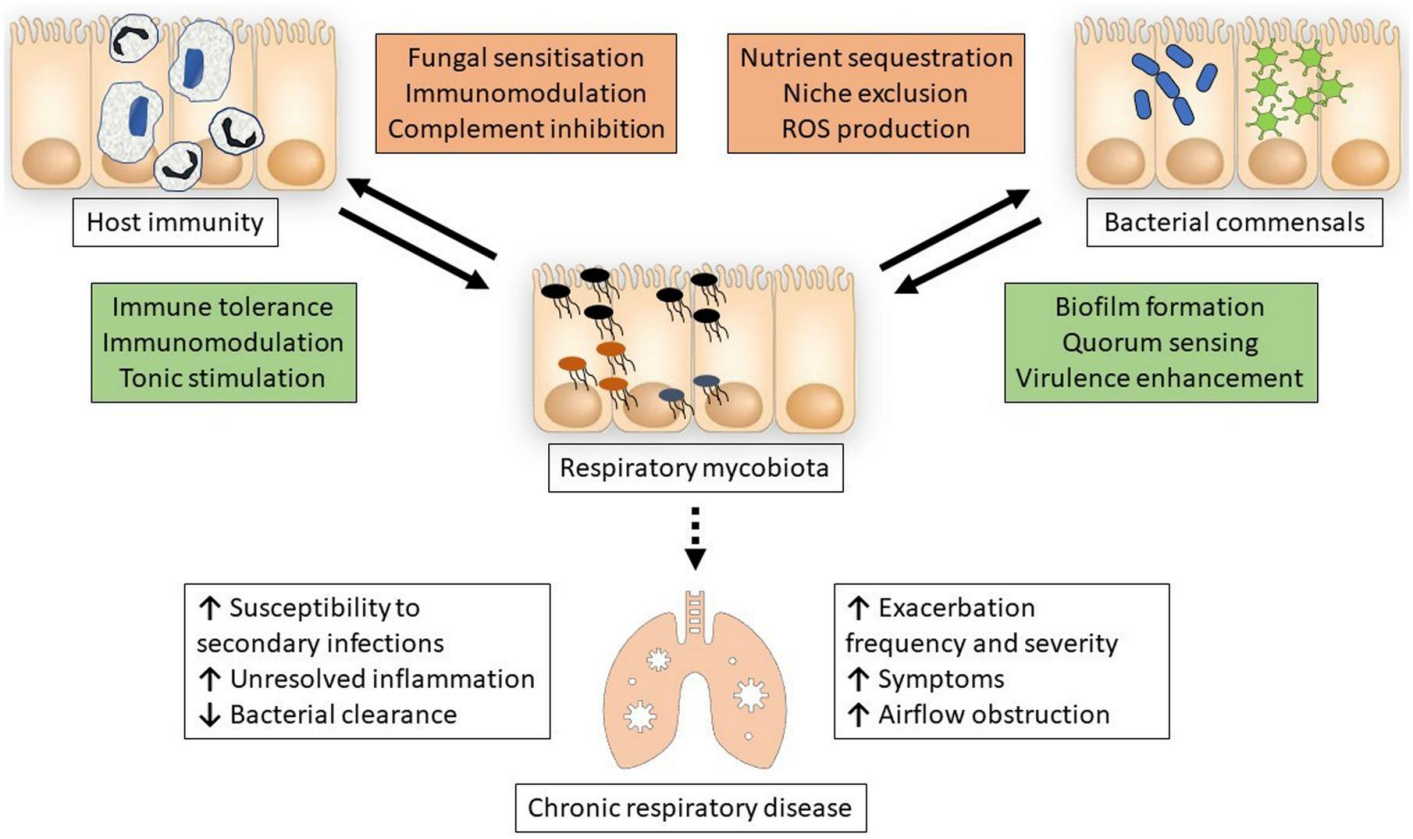
The complex interplay underlying the impact of the respiratory mycobiota on the development and progression of chronic respiratory diseases. Members of the respiratory mycobiota coinhabit the airways together with other commensal microorganisms. The inter-microbial relationships that exist between these groups can influence their growth and survival, and thus impact upon host immune homeostasis. Mycobiota can also directly manipulate host immunity via production of immunomodulatory metabolites and stimulation of host pattern recognition receptors. These interactions are particularly important in the context of chronic respiratory disease, where increasing evidence suggests that the composition of the respiratory mycobiota can impact the severity and frequency of disease exacerbations

Taken together the mycobiome appears to have roles in influencing the development and progression of CRDs. It is becoming increasingly apparent that the variety of geographical aspects, the genetic defects of the immune system, and the crosstalk between microorganisms of the wider microbiota, represent factors that could affect the clinical spectrum and the management of chronic respiratory diseases. The complex interactions between fungal and bacterial members of the respiratory microbiota and the host immune system are summarized in Fig. 2.

## Conclusion and future perspectives

The discovery of the human respiratory microbiome has led to profound questions around how commensals may impact upon immunity and disease pathogenesis. Despite significant advancements being made in molecular and sequencing technologies, the characterization of the fungal component of this microbial community remains incomplete. We are beginning to recognize that the composition of the healthy respiratory mycobiota differs significantly to that of CRDs. Even more gaps remain in our understanding of the functional effects of the respiratory mycobiota, and its involvement in the development and progression of various respiratory diseases. Increased understanding could be driven by improved assessment of microbial function through integrated omics technologies, more standardized experimental design, and a greater emphasis on functional experiments in tractable experimental models. It is also vital to recognize that the information pool on the interactions of commensal fungi with other bacterial and viral communities is currently very limited. Future research should thus also focus upon trans-kingdom interactions in this context. Table 2 summarizes research areas that may be of future interest to the field. Overall, the mycobiome is anticipated to play a significant role in health and disease and development of our understanding in this important field has the potential to drive novel therapeutic approaches.

**Table 2.** Outstanding research areas and questions that need to be addressed in the future, in order to further advance our understanding of the role of the airway mycobiome in the development and progression of chronic respiratory diseases (CRDs)

Research questions that remain to be addressed	Investigative approach
What are the key fungal species present in the airway mycobiome of healthy individuals? What is the airway mycobiome profile of various CRDs?	Comparative metagenomic analyses High-throughput ITS sequencing Standardized longitudinal cohort studies
Does dysbiosis of the mycobiome contribute to development of invasive or chronic fungal disease?	Longitudinal studies in immunosuppressed cohorts
Could airway mycobiome profiling be used as a diagnostic tool to predict CRD exacerbations in a clinical setting?	Characterization of airway mycobiome in health and disease context Development of accurate techniques for identification of fungi in various airway sample types
What are the functional roles played by different members of the airway mycobiome in maintaining immune homeostasis?	Metatranscriptomics and metabolomics studies Comparative studies with CRD patients Interventional experiments in CRD animal models
What are the interactions of members of the airway mycobiome with bacterial species not typically associated with overt respiratory infections?	Longitudinal correlational studies in CRD patients
How do environmental and lifestyle factors (e.g. pollution, allergens, smoking, diet) affect the composition and function of the airway mycobiome?	Epidemiological studies Longitudinal correlational studies encompassing geographical and lifestyle variation
How is the composition of the airway mycobiome influenced by the use of biologics therapies (e.g. monoclonal antibodies) for the treatment of type-2 asthma?	Interventional experiments in CRD animal models Characterization of airway mycobiome in human clinical trial patients
Can alterations in the composition of the airway mycobiome be used as biomarkers for the early detection of various respiratory conditions?	Characterization of airway mycobiome in health and disease contexts Validation of biomarker use in patient cohorts
What novel therapeutic strategies can be developed by manipulating the airway mycobiome in the context of various CRDs?	Interventional experiments in CRD animal models Clinical trials in human patients

## References

[1] Dickson RP , Erb-Downward JR, Martinez FJ, Huffnagle GB. The microbiome and the respiratory tract. Annu Rev Physiol 2016;78:481–504. 26527186 10.1146/annurev-physiol-021115-105238 PMC4751994

[2] Nguyen LD , Viscogliosi E, Delhaes L. The lung mycobiome: an emerging field of the human respiratory microbiome. Front Microbiol 2015;6:89. 25762987 10.3389/fmicb.2015.00089 PMC4327734

[3] Belvoncikova P , Splichalova P, Videnska P, Gardlik R. The human mycobiome: colonization, composition and the role in health and disease. J Fungi (Basel) 2022;8:1046. 36294611 10.3390/jof8101046 PMC9605233

[4] Findley K , Oh J, Yang J et al Topographic diversity of fungal and bacterial communities in human skin. Nature 2013;498:367–70. 23698366 10.1038/nature12171 PMC3711185

[5] Azevedo MJ , Pereira ML, Araujo R et al Influence of delivery and feeding mode in oral fungi colonization—a systematic review. Microb Cell 2020;7:36–45. 32025512 10.15698/mic2020.02.706 PMC6993125

[6] Wampach L , Heintz-Buschart A, Hogan A et al Colonization and succession within the human gut microbiome by archaea, bacteria, and microeukaryotes during the first year of life. Front Microbiol 2017;8:738. 28512451 10.3389/fmicb.2017.00738 PMC5411419

[7] David LA , Maurice CF, Carmody RN et al Diet rapidly and reproducibly alters the human gut microbiome. Nature 2014;505:559–63. 24336217 10.1038/nature12820 PMC3957428

[8] Belkaid Y , Hand TW. Role of the microbiota in immunity and inflammation. Cell 2014;157:121–41. 24679531 10.1016/j.cell.2014.03.011 PMC4056765

[9] Erb-Downward JR , Thompson DL, Han MK et al Analysis of the lung microbiome in the "healthy" smoker and in COPD. PloS One 2011;6:e16384. 21364979 10.1371/journal.pone.0016384 PMC3043049

[10] Morgan XC , Tickle TL, Sokol H et al Dysfunction of the intestinal microbiome in inflammatory bowel disease and treatment. Genome Biol 2012;13:R79. 23013615 10.1186/gb-2012-13-9-r79 PMC3506950

[11] Huffnagle GB , Noverr MC. The emerging world of the fungal microbiome. Trends Microbiol 2013;21:334–41. 23685069 10.1016/j.tim.2013.04.002 PMC3708484

[12] Chotirmall SH , Martin-Gomez MT. Aspergillus species in bronchiectasis: challenges in the cystic fibrosis and non-cystic fibrosis airways. Mycopathologia 2018;183:45–59. 28516246 10.1007/s11046-017-0143-7

[13] Tiew PY , Mac Aogain M, Ter SK et al Respiratory mycoses in COPD and bronchiectasis. Mycopathologia 2021;186:623–38. 33709335 10.1007/s11046-021-00539-z

[14] Jacobsen ID. The role of host and fungal factors in the commensal-to-pathogen transition of Candida albicans. Curr Clin Microbiol Rep 2023;10:55–65. 37151578 10.1007/s40588-023-00190-w PMC10154278

[15] Lai GC , Tan TG, Pavelka N. The mammalian mycobiome: a complex system in a dynamic relationship with the host. Wiley Interdiscip Rev Syst Biol Med 2019;11:e1438. 30255552 10.1002/wsbm.1438 PMC6586165

[16] Bignell E , Cairns TC, Throckmorton K et al Secondary metabolite arsenal of an opportunistic pathogenic fungus. Philos Trans R Soc Lond B Biol Sci 2016;371:20160023. 28080993 10.1098/rstb.2016.0023 PMC5095546

[17] Inglis DO , Binkley J, Skrzypek MS et al Comprehensive annotation of secondary metabolite biosynthetic genes and gene clusters of Aspergillus nidulans, A. fumigatus, A. Niger and A. oryzae. BMC Microbiol 2013;13:91. 23617571 10.1186/1471-2180-13-91 PMC3689640

[18] Steenwyk JL , Mead ME, Knowles SL et al Variation among biosynthetic gene clusters, secondary metabolite profiles, and cards of virulence across Aspergillus species. Genetics 2020;216:481–97. 32817009 10.1534/genetics.120.303549 PMC7536862

[19] Carpagnano GE , Susca A, Scioscia G et al A survey of fungal microbiota in airways of healthy volunteer subjects from Puglia (Apulia), Italy. BMC Infect Dis 2019;19:78. 30669978 10.1186/s12879-019-3718-8 PMC6341515

[20] Lücking R , Aime MC, Robbertse B et al Unambiguous identification of fungi: where do we stand and how accurate and precise is fungal DNA barcoding? IMA Fungus 2020;11:14. 32714773 10.1186/s43008-020-00033-z PMC7353689

[21] Richardson M , Bowyer P, Sabino R. The human lung and Aspergillus: you are what you breathe in? Med Mycol 2019;57:S145–S154. 30816978 10.1093/mmy/myy149 PMC6394755

[22] Frau A , Kenny JG, Lenzi L et al DNA extraction and amplicon production strategies deeply influence the outcome of gut mycobiome studies. Sci Rep 2019;9:9328. 31249384 10.1038/s41598-019-44974-x PMC6597572

[23] Witherden EA , Shoaie S, Hall RA, Moyes DL. The human mucosal mycobiome and fungal community interactions. J Fungi (Basel) 2017;3:56. 29371572 10.3390/jof3040056 PMC5753158

[24] Weaver D , Gago S, Bromley M, Bowyer P. The human lung mycobiome in chronic respiratory disease: limitations of methods and our current understanding. Curr Fungal Infect Rep 2019;13:109–19.

[25] Rolling T , Zhai B, Frame J et al Customization of a DADA2-based pipeline for fungal internal transcribed spacer 1 (ITS1) amplicon data sets. JCI Insight 2022;7:e151663. 10.1172/jci.insight.151663 PMC8765055 34813499

[26] Tang J , Iliev ID, Brown J et al Mycobiome: approaches to analysis of intestinal fungi. J Immunol Methods 2015;421:112–21. 25891793 10.1016/j.jim.2015.04.004 PMC4451377

[27] Tiew PY , Mac Aogain M, Ali NABM et al The mycobiome in health and disease: emerging concepts, methodologies and challenges. Mycopathologia 2020;185:207–31. 31894501 10.1007/s11046-019-00413-z PMC7223441

[28] Bharti R , Grimm DG. Current challenges and best-practice protocols for microbiome analysis. Brief Bioinform 2021;22:178–93. 31848574 10.1093/bib/bbz155 PMC7820839

[29] Schei K , Avershina E, Øien T et al Early gut mycobiota and mother-offspring transfer. Microbiome 2017;5:107. 28837002 10.1186/s40168-017-0319-x PMC5571498

[30] Strati F , Di Paola M, Stefanini I et al Age and gender affect the composition of fungal population of the human gastrointestinal tract. Front Microbiol 2016;7:1227. 27536299 10.3389/fmicb.2016.01227 PMC4971113

[31] Huffnagle GB , Dickson RP, Lukacs NW. The respiratory tract microbiome and lung inflammation: a two-way street. Mucosal Immunol 2017;10:299–306. 27966551 10.1038/mi.2016.108 PMC5765541

[32] Dickson RP , Huffnagle GB. The lung microbiome: new principles for respiratory bacteriology in health and disease. PLoS Pathog 2015;11:e1004923. 26158874 10.1371/journal.ppat.1004923 PMC4497592

[33] Soret P , Vandenborght L-E, Francis F et al Respiratory mycobiome and suggestion of inter-kingdom network during acute pulmonary exacerbation in cystic fibrosis. Sci Rep 2020;10:3589. 32108159 10.1038/s41598-020-60015-4 PMC7046743

[34] Cui L , Lucht L, Tipton L et al Topographic diversity of the respiratory tract mycobiome and alteration in HIV and lung disease. Am J Respir Crit Care Med 2015;191:932–42. 25603113 10.1164/rccm.201409-1583OC PMC4435454

[35] Nash AK , Auchtung TA, Wong MC et al The gut mycobiome of the Human Microbiome Project healthy cohort. Microbiome 2017;5:153. 29178920 10.1186/s40168-017-0373-4 PMC5702186

[36] Narayana JK , Aliberti S, Mac Aogáin M et al Microbial dysregulation of the gut-lung axis in bronchiectasis. Am J Respir Crit Care Med 2023;207:908–20. 36288294 10.1164/rccm.202205-0893OC PMC10111978

[37] Rozaliyani A , Antariksa B, Nurwidya F et al The fungal and bacterial interface in the respiratory mycobiome with a focus on Aspergillus spp. Life (Basel) 2023;13:1017. 37109545 10.3390/life13041017 PMC10142979

[38] Monteiro-da-Silva F , Araujo R, Sampaio-Maia B. Interindividual variability and intraindividual stability of oral fungal microbiota over time. Med Mycol 2014;52:498–505. 24934804 10.1093/mmy/myu027

[39] Oba PM , Holscher HD, Mathai RA et al Diet influences the oral microbiota of infants during the first six months of life. Nutrients 2020;12:3400. 33167488 10.3390/nu12113400 PMC7694519

[40] Ghannoum MA , Jurevic RJ, Mukherjee PK et al Characterization of the oral fungal microbiome (mycobiome) in healthy individuals. PLoS Pathog 2010;6:e1000713. 20072605 10.1371/journal.ppat.1000713 PMC2795202

[41] Underhill DM , Iliev ID. The mycobiota: interactions between commensal fungi and the host immune system. Nat Rev Immunol 2014;14:405–16. 24854590 10.1038/nri3684 PMC4332855

[42] Peters BA , Wu J, Hayes RB, Ahn J. The oral fungal mycobiome: characteristics and relation to periodontitis in a pilot study. BMC Microbiol 2017;17:157. 28701186 10.1186/s12866-017-1064-9 PMC5508751

[43] Dupuy AK , David MS, Li L et al Redefining the human oral mycobiome with improved practices in amplicon-based taxonomy: discovery of Malassezia as a prominent commensal. PloS One 2014;9:e90899. 24614173 10.1371/journal.pone.0090899 PMC3948697

[44] Diaz PI , Dongari-Bagtzoglou A. Critically appraising the significance of the oral mycobiome. J Dent Res 2021;100:133–40. 32924741 10.1177/0022034520956975 PMC8173349

[45] Fechney JM , Browne GV, Prabhu N et al Preliminary study of the oral mycobiome of children with and without dental caries. J Oral Microbiol 2019;11:1536182. 30598729 10.1080/20002297.2018.1536182 PMC6225480

[46] O'Connell LM , Santos R, Springer G et al Site-specific profiling of the dental mycobiome reveals strong taxonomic shifts during progression of early-childhood caries. Appl Environ Microbiol 2020;86:e02825–19. 31953340 10.1128/AEM.02825-19 PMC7082576

[47] Mukherjee PK , Chandra J, Retuerto M et al Oral mycobiome analysis of HIV-infected patients: identification of Pichia as an antagonist of opportunistic fungi. PLoS Pathog 2014;10:e1003996. 24626467 10.1371/journal.ppat.1003996 PMC3953492

[48] Mac Aogáin M , Chandrasekaran R, Lim AYH et al Immunological corollary of the pulmonary mycobiome in bronchiectasis: the CAMEB study. Eur Respir J 2018;52:1800766. 29880655 10.1183/13993003.00766-2018 PMC6092680

[49] Martinsen EMH , Eagan TML, Leiten EO et al The pulmonary mycobiome-a study of subjects with and without chronic obstructive pulmonary disease. PloS One 2021;16:e0248967. 33826639 10.1371/journal.pone.0248967 PMC8026037

[50] Rick E-M , Woolnough KF, Seear PJ et al The airway fungal microbiome in asthma. Clin Exp Allergy 2020;50:1325–41. 32808353 10.1111/cea.13722

[51] Tiew PY , Dicker AJ, Keir HR et al A high-risk airway mycobiome is associated with frequent exacerbation and mortality in COPD. Eur Respir J 2021;57:2002050. 32972986 10.1183/13993003.02050-2020

[52] van Woerden HC , Gregory C, Brown R et al Differences in fungi present in induced sputum samples from asthma patients and non-atopic controls: a community based case control study. BMC Infect Dis 2013;13:69. 23384395 10.1186/1471-2334-13-69 PMC3570489

[53] Ritchie AI , Singanayagam A. Metagenomic characterization of the respiratory microbiome: a piece de resistance. Am J Respir Crit Care Med 2020;202:321–2. 32442018 10.1164/rccm.202005-1686ED PMC7397782

[54] Brown RL , Sequeira RP, Clarke TB. The microbiota protects against respiratory infection via GM-CSF signaling. Nat Commun 2017;8:1512. 29142211 10.1038/s41467-017-01803-x PMC5688119

[55] Honda K , Littman DR. The microbiome in infectious disease and inflammation. Annu Rev Immunol 2012;30:759–95. 22224764 10.1146/annurev-immunol-020711-074937 PMC4426968

[56] Jiang TT , Shao T-Y, Ang WXG et al Commensal fungi recapitulate the protective benefits of intestinal bacteria. Cell Host Microbe 2017;22:809–16 e4. 29174402 10.1016/j.chom.2017.10.013 PMC5730478

[57] Magiatis P , Pappas P, Gaitanis G et al Malassezia yeasts produce a collection of exceptionally potent activators of the Ah (dioxin) receptor detected in diseased human skin. J Invest Dermatol 2013;133:2023–30. 23448877 10.1038/jid.2013.92 PMC3714356

[58] Vlachos C , Schulte BM, Magiatis P et al Malassezia-derived indoles activate the aryl hydrocarbon receptor and inhibit Toll-like receptor-induced maturation in monocyte-derived dendritic cells. Br J Dermatol 2012;167:496–505. 22533375 10.1111/j.1365-2133.2012.11014.x

[59] Kim CH. Control of lymphocyte functions by gut microbiota-derived short-chain fatty acids. Cell Mol Immunol 2021;18:1161–71. 33850311 10.1038/s41423-020-00625-0 PMC8093302

[60] Kim YG , Udayanga KG, Totsuka N et al Gut dysbiosis promotes M2 macrophage polarization and allergic airway inflammation via fungi-induced PGE(2). Cell Host Microbe 2014;15:95–102. 24439901 10.1016/j.chom.2013.12.010 PMC3957200

[61] Baltierra-Trejo E , Sánchez-Yáñez JM, Buenrostro-Delgado O, Márquez-Benavides L. Production of short-chain fatty acids from the biodegradation of wheat straw lignin by Aspergillus fumigatus. Bioresour Technol 2015;196:418–25. 26263005 10.1016/j.biortech.2015.07.105

[62] Quintin J , Saeed S, Martens JHA et al Candida albicans infection affords protection against reinfection via functional reprogramming of monocytes. Cell Host Microbe 2012;12:223–32. 22901542 10.1016/j.chom.2012.06.006 PMC3864037

[63] Netea MG , Sutmuller R, Hermann C et al Toll-like receptor 2 suppresses immunity against Candida albicans through induction of IL-10 and regulatory T cells. J Immunol 2004;172:3712–8. 15004175 10.4049/jimmunol.172.6.3712

[64] Navarathna DH , Nickerson KW, Duhamel GE et al Exogenous farnesol interferes with the normal progression of cytokine expression during candidiasis in a mouse model. Infect Immun 2007;75:4006–11. 17517874 10.1128/IAI.00397-07 PMC1951970

[65] Krupa A , Kowalska I. The kynurenine pathway-new linkage between innate and adaptive immunity in autoimmune endocrinopathies. Int J Mol Sci 2021;22:9879. 34576041 10.3390/ijms22189879 PMC8469440

[66] Romani L , Zelante T, De Luca A et al Microbiota control of a tryptophan-AhR pathway in disease tolerance to fungi. Eur J Immunol 2014;44:3192–200. 25256754 10.1002/eji.201344406

[67] Zhang I , Pletcher SD, Goldberg AN et al Fungal microbiota in chronic airway inflammatory disease and emerging relationships with the host immune response. Front Microbiol 2017;8:2477. 29312187 10.3389/fmicb.2017.02477 PMC5733051

[68] Morton R , Singanayagam A. The respiratory tract microbiome: moving from correlation to causation. Eur Respir J 2022;59. 10.1183/13993003.03079-2021 35512808

[69] Mac Aogáin M , Narayana JK, Tiew PY et al Integrative microbiomics in bronchiectasis exacerbations. Nat Med 2021;27:688–99. 33820995 10.1038/s41591-021-01289-7

[70] Sanderson AR , Leid JG, Hunsaker D. Bacterial biofilms on the sinus mucosa of human subjects with chronic rhinosinusitis. Laryngoscope 2006;116:1121–6. 16826045 10.1097/01.mlg.0000221954.05467.54

[71] Peleg AY , Hogan DA, Mylonakis E. Medically important bacterial-fungal interactions. Nat Rev Microbiol 2010;8:340–9. 20348933 10.1038/nrmicro2313

[72] Ferreira JAG , Penner JC, Moss RB et al Inhibition of Aspergillus fumigatus and its biofilm by Pseudomonas aeruginosa is dependent on the source, phenotype and growth conditions of the bacterium. PloS One 2015;10:e0134692. 26252384 10.1371/journal.pone.0134692 PMC4529298

[73] Briard B , Bomme P, Lechner BE et al Pseudomonas aeruginosa manipulates redox and iron homeostasis of its microbiota partner Aspergillus fumigatus via phenazines. Sci Rep 2015;5:8220. 25665925 10.1038/srep08220 PMC5389140

[74] Penner JC , Ferreira JAG, Secor PR et al Pf4 bacteriophage produced by Pseudomonas aeruginosa inhibits Aspergillus fumigatus metabolism via iron sequestration. Microbiology (Reading) 2016;162:1583–94. 27473221 10.1099/mic.0.000344

[75] Xu X-L , Lee RTH, Fang H-M et al Bacterial peptidoglycan triggers Candida albicans hyphal growth by directly activating the adenylyl cyclase Cyr1p. Cell Host Microbe 2008;4:28–39. 18621008 10.1016/j.chom.2008.05.014

[76] Adam B , Baillie GS, Douglas LJ. Mixed species biofilms of Candida albicans and Staphylococcus epidermidis. J Med Microbiol 2002;51:344–9. 11926741 10.1099/0022-1317-51-4-344

[77] Dixon EF , Hall RA. Noisy neighbourhoods: quorum sensing in fungal-polymicrobial infections. Cell Microbiol 2015;17:1431–41. 26243526 10.1111/cmi.12490 PMC4973845

[78] Rodrigues CF , Černáková L. Farnesol and tyrosol: secondary metabolites with a crucial quorum-sensing role in candida biofilm development. Genes (Basel) 2020;11:444. 32325685 10.3390/genes11040444 PMC7231263

[79] de Dios Caballero J , Cantón R, Ponce-Alonso M et al The human mycobiome in chronic respiratory diseases: current situation and future perspectives. Microorganisms 2022;10:810. 35456861 10.3390/microorganisms10040810 PMC9029612

[80] Tiew PY , Thng KX, Chotirmall SH. Clinical Aspergillus signatures in COPD and bronchiectasis. J Fungi (Basel) 2022;8:480. 35628736 10.3390/jof8050480 PMC9146266

[81] Al Shakirchi M , Sorjonen K, Klingspor L et al The effects of Aspergillus fumigatus colonization on lung function in patients with cystic fibrosis. J Fungi (Basel) 2021;7:944. 34829231 10.3390/jof7110944 PMC8618016

[82] Barnes PJ , Burney PGJ, Silverman EK et al Chronic obstructive pulmonary disease. Nat Rev Dis Primers 2015;1:15076. 27189863 10.1038/nrdp.2015.76

[83] Singanayagam A , Loo S-L, Calderazzo M et al Antiviral immunity is impaired in COPD patients with frequent exacerbations. Am J Physiol Lung Cell Mol Physiol 2019;317:L893–L903. 31513433 10.1152/ajplung.00253.2019 PMC6962603

[84] Connell D , Shah A. The contribution of Aspergillus fumigatus to COPD exacerbations: a "sensitive" topic. Eur Respir J 2020;56.2002223. 32855304 10.1183/13993003.02223-2020

[85] Singanayagam A , Glanville N, Girkin JL et al Corticosteroid suppression of antiviral immunity increases bacterial loads and mucus production in COPD exacerbations. Nat Commun 2018;9:2229. 29884817 10.1038/s41467-018-04574-1 PMC5993715

[86] Su J , Liu H-y, Tan X-L et al Sputum bacterial and fungal dynamics during exacerbations of severe COPD. PloS One 2015;10:e0130736. 26147303 10.1371/journal.pone.0130736 PMC4493005

[87] Enaud R , Sioniac P, Imbert S et al Lung mycobiota alpha-diversity is linked to severity in critically ill patients with acute exacerbation of chronic obstructive pulmonary disease. Microbiol Spectr 2023;11:e0506222. 36976010 10.1128/spectrum.05062-22 PMC10100765

[88] Bafadhel M , McKenna S, Agbetile J et al Aspergillus fumigatus during stable state and exacerbations of COPD. Eur Respir J 2014;43:64–71. 23598955 10.1183/09031936.00162912

[89] Hammond EE , McDonald CS, Vestbo J, Denning DW. The global impact of Aspergillus infection on COPD. BMC Pulm Med 2020;20:241. 32912168 10.1186/s12890-020-01259-8 PMC7488557

[90] Tong X , Cheng A, Xu H et al Aspergillus fumigatus during COPD exacerbation: a pair-matched retrospective study. BMC Pulm Med 2018;18:55. 29615101 10.1186/s12890-018-0611-y PMC5883425

[91] Urb M , Snarr BD, Wojewodka G et al Evolution of the immune response to chronic airway colonization with Aspergillus fumigatus hyphae. Infect Immun 2015;83:3590–600. 26123803 10.1128/IAI.00359-15 PMC4534667

[92] Chaudhary N , Marr KA. Impact of Aspergillus fumigatus in allergic airway diseases. Clin Transl Allergy 2011;1:4. 22410255 10.1186/2045-7022-1-4 PMC3294627

[93] Wu Y-X , Zuo Y-H, Cheng Q-J et al Respiratory Aspergillus colonization was associated with relapse of acute exacerbation in patients with chronic obstructive pulmonary disease: analysis of data from a retrospective cohort study. Front Med (Lausanne) 2021;8:640289. 34017841 10.3389/fmed.2021.640289 PMC8129169

[94] Zuo Y-H , Wang W-Q, Chen Q-J et al Candida in lower respiratory tract increases the frequency of acute exacerbation of chronic obstructive pulmonary disease: a retrospective case-control study. Front Cell Infect Microbiol 2020;10:538005. 33117725 10.3389/fcimb.2020.538005 PMC7561360

[95] Huerta A , Soler N, Esperatti M et al Importance of Aspergillus spp. isolation in acute exacerbations of severe COPD: prevalence, factors and follow-up: the FUNGI-COPD study. Respir Res 2014;15:17. 24517318 10.1186/1465-9921-15-17 PMC3996133

[96] Liu H , Liang Z, Cao N et al Airway bacterial and fungal microbiome in chronic obstructive pulmonary disease. Medicine in Microecology 2021;7:100035.

[97] Brightling CE , Monteiro W, Ward R et al Sputum eosinophilia and short-term response to prednisolone in chronic obstructive pulmonary disease: a randomised controlled trial. Lancet 2000;356:1480–5. 11081531 10.1016/S0140-6736(00)02872-5

[98] Yang R , Zhang Q, Ren Z et al Different airway inflammatory phenotypes correlate with specific fungal and bacterial microbiota in asthma and chronic obstructive pulmonary disease. J Immunol Res 2022;2022:2177884. 35310604 10.1155/2022/2177884 PMC8933093

[99] He M , Ichinose T, Song Y et al The role of toll-like receptors and myeloid differentiation factor 88 in bjerkandera adusta-induced lung inflammation. Int Arch Allergy Immunol 2015;168:96–106. 26641462 10.1159/000441895

[100] He M , Ichinose T, Liu B et al Silica-carrying particulate matter enhances Bjerkandera adusta-induced murine lung eosinophilia. Environ Toxicol 2016;31:93–105. 25044538 10.1002/tox.22025

[101] Denis O , van den Brûle S, Heymans J et al Chronic intranasal administration of mould spores or extracts to unsensitized mice leads to lung allergic inflammation, hyper-reactivity and remodelling. Immunology 2007;122:268–78. 17506853 10.1111/j.1365-2567.2007.02636.x PMC2265999

[102] Agarwal K , Gaur SN, Chowdhary A. The role of fungal sensitisation in clinical presentation in patients with chronic obstructive pulmonary disease. Mycoses 2015;58:531–5. 26201384 10.1111/myc.12352

[103] Canas-Arboleda A , Hernandez-Florez C, Garzon J et al Colonization by Pneumocystis jirovecii in patients with chronic obstructive pulmonary disease: association with exacerbations and lung function status. Braz J Infect Dis 2019;23:352–7. 31545952 10.1016/j.bjid.2019.08.008 PMC9427795

[104] Morris A , Sciurba FC, Lebedeva IP et al Association of chronic obstructive pulmonary disease severity and Pneumocystis colonization. Am J Respir Crit Care Med 2004;170:408–13. 15117741 10.1164/rccm.200401-094OC

[105] Fitzpatrick ME , Tedrow JR, Hillenbrand ME et al Pneumocystis jirovecii colonization is associated with enhanced Th1 inflammatory gene expression in lungs of humans with chronic obstructive pulmonary disease. Microbiol Immunol 2014;58:202–11. 24438206 10.1111/1348-0421.12135 PMC4106795

[106] Gantois N , Lesaffre A, Durand-Joly I et al Factors associated with Pneumocystis colonization and circulating genotypes in chronic obstructive pulmonary disease patients with acute exacerbation or at stable state and their homes. Med Mycol 2021;60:myab070. 10.1093/mmy/myab070 34734270

[107] Xue T , Ma Z, Liu F et al Pneumocystis jirovecii colonization and its association with pulmonary diseases: a multicenter study based on a modified loop-mediated isothermal amplification assay. BMC Pulm Med 2020;20:70. 32197601 10.1186/s12890-020-1111-4 PMC7085144

[108] Singanayagam A , Ritchie AI, Johnston SL. Role of microbiome in the pathophysiology and disease course of asthma. Curr Opin Pulm Med 2017;23:41–7. 27755161 10.1097/MCP.0000000000000333

[109] Holgate ST , Wenzel S, Postma DS et al Asthma. Nat Rev Dis Primers 2015;1:15025. 27189668 10.1038/nrdp.2015.25 PMC7096989

[110] Maule M , Vitte J, Ambrosani F, Caminati M. Epidemiology of the relationship between allergic bronchopulmonary aspergillosis and asthma. Curr Opin Allergy Clin Immunol 2024;24:102–88. 38295145 10.1097/ACI.0000000000000971

[111] Pashley CH , Wardlaw AJ. Allergic fungal airways disease (AFAD): an under-recognised asthma endotype. Mycopathologia 2021;186:609–22. 34043134 10.1007/s11046-021-00562-0 PMC8536613

[112] Huang C , Yu Y, Du W et al Fungal and bacterial microbiome dysbiosis and imbalance of trans-kingdom network in asthma. Clin Transl Allergy 2020;10:42. 33110490 10.1186/s13601-020-00345-8 PMC7583303

[113] Goh KJ , Yii ACA, Lapperre TS et al Sensitization to Aspergillus species is associated with frequent exacerbations in severe asthma. J Asthma Allergy 2017;10:131–40. 28461762 10.2147/JAA.S130459 PMC5407445

[114] Fraczek MG , Chishimba L, Niven RM et al Corticosteroid treatment is associated with increased filamentous fungal burden in allergic fungal disease. J Allergy Clin Immunol 2018;142:407–14. 29122659 10.1016/j.jaci.2017.09.039

[115] Sharma A , Laxman B, Naureckas ET et al Associations between fungal and bacterial microbiota of airways and asthma endotypes. J Allergy Clin Immunol 2019;144:1214–27 e7. 31279011 10.1016/j.jaci.2019.06.025 PMC6842419

[116] Xu X , Ding F, Hu X et al Upper respiratory tract mycobiome alterations in different kinds of pulmonary disease. Front Microbiol 2023;14:1117779. 37032908 10.3389/fmicb.2023.1117779 PMC10076636

[117] Yuan H , Liu Z, Dong J et al The fungal microbiome of the upper airway is associated with future loss of asthma control and exacerbation among children with asthma. Chest 2023;164:302–13. 37003356 10.1016/j.chest.2023.03.034 PMC10477953

[118] Sharpe RA , Bearman N, Thornton CR et al Indoor fungal diversity and asthma: a meta-analysis and systematic review of risk factors. J Allergy Clin Immunol 2015;135:110–22. 25159468 10.1016/j.jaci.2014.07.002

[119] Noverr MC , Noggle RM, Toews GB, Huffnagle GB. Role of antibiotics and fungal microbiota in driving pulmonary allergic responses. Infect Immun 2004;72:4996–5003. 15321991 10.1128/IAI.72.9.4996-5003.2004 PMC517468

[120] Ratjen F , Bell SC, Rowe SM et al Cystic fibrosis. Nat Rev Dis Primers 2015;1:15010. 27189798 10.1038/nrdp.2015.10 PMC7041544

[121] Blanchard AC , Waters VJ. Opportunistic pathogens in cystic fibrosis: epidemiology and pathogenesis of lung infection. J Pediatric Infect Dis Soc 2022;11:S3–S12. 36069904 10.1093/jpids/piac052

[122] Delhaes L , Monchy S, Fréalle E et al The airway microbiota in cystic fibrosis: a complex fungal and bacterial community—implications for therapeutic management. PloS One 2012;7:e36313. 22558432 10.1371/journal.pone.0036313 PMC3338676

[123] Cuthbertson L , Felton I, James P et al The fungal airway microbiome in cystic fibrosis and non-cystic fibrosis bronchiectasis. J Cyst Fibros 2021;20:295–302. 32540174 10.1016/j.jcf.2020.05.013 PMC8048771

[124] Kramer R , Sauer-Heilborn A, Welte T et al Cohort study of airway mycobiome in adult cystic fibrosis patients: differences in community structure between fungi and bacteria reveal predominance of transient fungal elements. J Clin Microbiol 2015;53:2900–7. 26135861 10.1128/JCM.01094-15 PMC4540938

[125] Willger SD , Grim SL, Dolben EL et al Characterization and quantification of the fungal microbiome in serial samples from individuals with cystic fibrosis. Microbiome 2014;2:40. 25408892 10.1186/2049-2618-2-40 PMC4236224

[126] Duesberg U , Wosniok J, Naehrlich L et al Risk factors for respiratory Aspergillus fumigatus in German Cystic Fibrosis patients and impact on lung function. Sci Rep 2020;10:18999. 33149181 10.1038/s41598-020-75886-w PMC7643137

[127] O'Connor JB , Wagner BD, Harris JK et al Detection and identification of fungi in the lower airway of children with and without cystic fibrosis. Front Microbiol 2023;14:1119703. 36846802 10.3389/fmicb.2023.1119703 PMC9948248

[128] Warris A , Bercusson A, Armstrong-James D. Aspergillus colonization and antifungal immunity in cystic fibrosis patients. Med Mycol 2019;57:S118–S126. 30816976 10.1093/mmy/myy074

[129] Bargon J , Dauletbaev N, Kohler B et al Prophylactic antibiotic therapy is associated with an increased prevalence of Aspergillus colonization in adult cystic fibrosis patients. Respir Med 1999;93:835–8. 10603634 10.1016/s0954-6111(99)90270-6

[130] Agarwal R , Muthu V, Sehgal IS. Clinical manifestation and treatment of allergic bronchopulmonary Aspergillosis. Semin Respir Crit Care Med 2024;45:114–27. 38154470 10.1055/s-0043-1776912

